# Association between extracellular-to-total body water ratio and eGFR decline in diabetic kidney disease: a longitudinal cohort study

**DOI:** 10.1007/s10157-026-02905-x

**Published:** 2026-06-19

**Authors:** Shotaro Miyamoto, Nao Imaishi, Machiko Morita, Hiroki Uchida, Yuko Yamazaki, Miyoko Osue, Yuko Hirota, Mitsuhiro Okamoto, Satoshi Nagai, Kentaro Sada, Naoki Matsuda, Takaaki Noguchi, Yoshinori Ozeki, Takeshi Nakata, Yuichi Yoshida, Akihiro Fukuda, Koro Gotoh, Takayuki Masaki, Hirotaka Shibata

**Affiliations:** 1 Department of Endocrinology, Metabolism, Rheumatology and Nephrology, Faculty of Medicine, Oita University, Yufu, Oita 879-5593 Japan; 2 Diabetes Kidney Disease Prevention Outpatient Program, Oita University Hospital, Oita University School of Medicine, Yufu, 879-5593 Japan; 3 Nursing Department, Oita University Hospital, Oita University School of Medicine, Yufu, 879-5593 Japan; 4 Clinical Nutrition Management Office, Oita University Hospital, Oita University School of Medicine, Yufu, 879-5593 Japan; 5 Abe Diabetes Clinic, 16-13 Nakakasugamachi, , Oita, 870-0039 Japan; 6 Faculty of Welfare and Health Sciences, Oita University, Oita, 870-1192 Japan; 7 Research Center for Global and Local Infectious Diseases, Oita University, Yufu, 879-5593 Japan; 8 Geriatric Nursing, Department of Nursing, Faculty of Medicine, Oita University, Yufu, 879-5593 Japan

**Keywords:** Diabetic kidney disease, Extracellular-to-total body water ratio, Bioelectrical impedance analysis, eGFR decline, Volume overload, Albuminuria, SGLT2 inhibitor

## Abstract

**Background:**

Diabetic kidney disease (DKD) can progress even in the absence of overt albuminuria, highlighting the need for markers for evaluating nonglomerular pathways. The extracellular-to-total body water ratio (ECW/TBW), measured via bioelectrical impedance analysis (BIA), reflects systemic fluid status; however, its longitudinal relevance to DKD progression remains unclear to date.

**Methods:**

We conducted a retrospective observational cohort study using longitudinal outpatient data from October 2020 to April 2025 at the Diabetic Kidney Disease Prevention Clinic, Oita University Hospital. A total of 40 adults with type 2 diabetes and clinically diagnosed DKD contributed 355 outpatient visits. The primary outcome was the annual rate of decline in estimated glomerular filtration rate (eGFR slope, mL/min/1.73 m^2^/year).

**Results:**

Both baseline and time-averaged ECW/TBW values were negatively correlated with eGFR slope in the univariate analysis (*p* = 0.014 and 0.025, respectively) and remained significant in the multivariate models. Log-transformed urinary albumin-to-creatinine ratio (UACR) was also significantly associated with faster eGFR decline. Treatment with sodium–glucose cotransporter 2 inhibitors (SGLT2is) was associated with lower ECW/TBW values, whereas other renoprotective medications exerted more modest individual effects, with greater reductions observed with the concurrent use of multiple agents.

**Conclusions:**

Higher ECW/TBW values were associated with faster eGFR decline in DKD, suggesting that subclinical volume overload may represent a potential nonglomerular mechanism of disease progression. This volume-related burden may be attenuated by SGLT2is therapy.

**Supplementary Information:**

The online version contains supplementary material available at https://doi.org/10.1007/s10157-026-02905-x.

## Introduction

Diabetic kidney disease (DKD) is a leading cause of renal replacement therapy and cardiovascular mortality [1]. While albuminuria and reduced glomerular filtration rate (GFR) remain central to the staging of DKD [2], many patients experience renal decline without overt albuminuria, a condition known as nonalbuminuric DKD [3, 4]. This condition indicates the involvement of alternative nonglomerular mechanisms beyond those reflected by albuminuria and GFR.

DKD has traditionally been attributed to a combination of metabolic and hemodynamic mechanisms. Chronic hyperglycemia promotes metabolic disturbances, including accumulation of advanced glycation end-products [5], polyol pathway activation [6], and protein kinase C signaling [7], which trigger oxidative stress and inflammation [8]. Hemodynamic factors, such as glomerular hyperfiltration and RAS activation [9], further contribute to podocyte damage [10] and albuminuria. In the tubulointerstitial compartment, protein overload, ischemia, and lipotoxicity trigger inflammation and fibrosis, leading to progressive nephron loss [11].

In diabetes, several factors beyond DKD itself contribute to fluid retention, including insulin-mediated enhanced renal sodium reabsorption [12] and concomitant heart failure. In particular, subclinical or undiagnosed heart failure is common among patients with diabetes [13, 14].

From a mechanistic perspective, volume overload can potentially exacerbate kidney injury by increasing renal venous pressure, a well-known concept in cardiorenal syndrome [15]. The unique renal vascular architecture makes it particularly vulnerable to even mild elevations in venous pressure [16]. However, the impact of systemic fluid retention on DKD progression has not been established.

Extracellular-to-total body water ratio (ECW/TBW), which is measured noninvasively by bioelectrical impedance analysis (BIA), quantitatively describes body–fluid distribution. Higher ECW/TBW values have been reported in ocular microvascular complications of diabetes, particularly proliferative diabetic retinopathy and diabetic macular edema [17–19]. However, the relevance of ECW/TBW values to DKD remains largely unexplored. Only one prior study has demonstrated an association between baseline ECW/TBW and faster CKD progression in type 2 diabetes. However, it only categorized the observed progression and did not analyze eGFR slope or serial ECW/TBW measurements [20], thus leaving its longitudinal relationship with renal decline unclear.

Therefore, we investigated the implications of persistently elevated ECW/TBW, as assessed through continuous BIA measurements, on eGFR decline in patients with DKD.

## Materials and methods

### Study design and population

This retrospective observational cohort study analyzed the longitudinal data from 355 outpatient visits of 40 adults with diabetic kidney disease.

Initially, 72 patients with suspected DKD were referred from other hospitals to the DKD Prevention Clinic of Oita University Hospital between October 2020 and April 2025. Patients were eligible for inclusion if they had at least 3 outpatient visits during the observation period. Patients were excluded if they had fewer than three visits, if renal decline was attributed to nondiabetic kidney disease, or if BIA could not be performed.

**Fig. 1:**
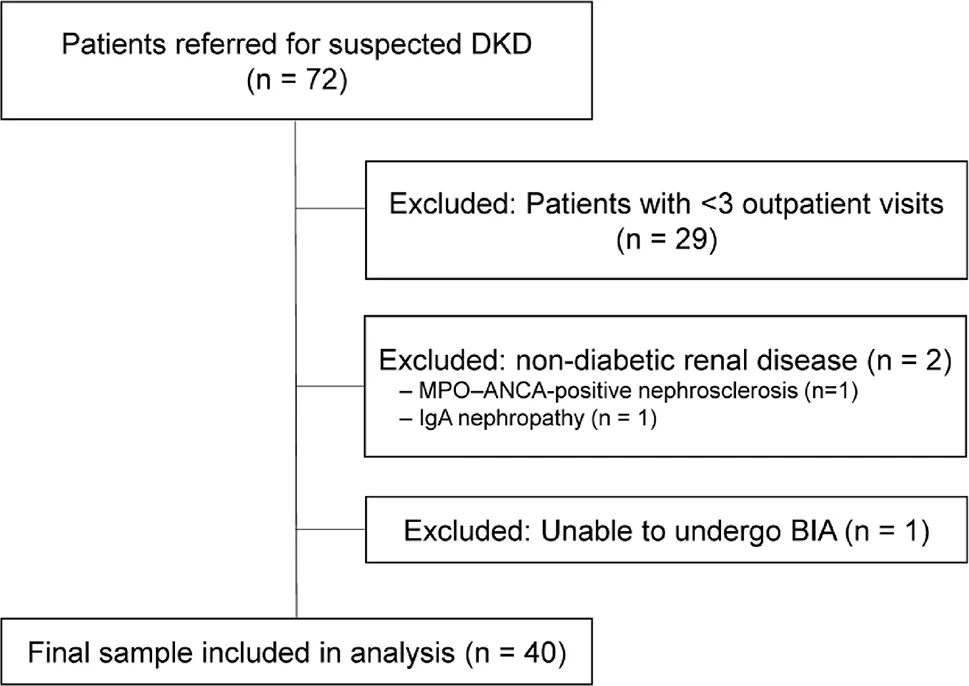
Flow diagram of patient inclusion and exclusion. *For five patients, who later shifted their primary diabetes care to our hospital and were subsequently included in the analysis, only the data obtained before this transition were used.* Alt text* Flow diagram illustrating the inclusion and exclusion process used to select patients for the final study cohort

Based on these criteria, twenty-nine patients were excluded due to insufficient follow-up (< 3 visits). Two more patients were ruled out because their renal decline was attributed to nondiabetic kidney diseases, one due to immunoglobulin A nephropathy and the other due to positivity for myeloperoxidase–antineutrophil cytoplasmic antibody. One patient was excluded because BIA could not be performed at any visit. Ultimately, 40 patients were included in the final analysis (Fig. 1).

At each visit, standardized assessments including serum creatinine, cystatin C, urinary albumin-to-creatinine ratio (UACR), glycated hemoglobin (HbA1c), and BIA were routinely performed. Additional clinical and laboratory parameters were also collected as part of routine care. Follow-up duration and visit intervals were not standardized, reflecting routine clinical practice, and some patients had relatively short observation periods. DKD was defined clinically as the presence of albuminuria, as indicated by a UACR ≥ 30 mg/gCr, eGFR decline (< 60 mL/min/1.73 m^2^), or both, in accordance with the guidelines of the 2022 Kidney Disease: Improving Global Outcomes (KDIGO) [21].

The study was approved by the Ethics Committee of Oita University Hospital (approval number 3,187). Informed consent was obtained through an opt-out process on the institution’s website, in accordance with the ethical guidelines for medical research involving human subjects in Japan.

### Clinical setting and outcome measures

The primary endpoint was the eGFR slope (mL/min/1.73 m^2^/year). eGFR was calculated from serum creatinine levels, using the equation established by the Japanese Society of Nephrology [22]: eGFR = 194 × serum creatinine^–1.094^ × age^–0.287^ (× 0.739 if female). As an exploratory analysis, the discrepancy between creatinine-based eGFR and cystatin C–based eGFR (eGFRdiff = eGFRcys – eGFRcr) was calculated [23]. The ECW/TBW ratio was analyzed as the primary exposure variable. UACR (mg/gCr), urinary protein (g/gCr), and estimated salt intake were also recorded.

### Bioelectrical impedance analysis

Body composition was assessed using a BIA device (InBody 770, Biospace Co., Ltd., Tokyo, Japan), following standardized standing-position procedures. The device recorded segmental impedance measurements to evaluate body composition. All BIA measurements were performed in the afternoon using the same device at our clinic. For this study, ECW, intracellular water (ICW), TBW, and the ECW/TBW ratio were extracted longitudinally. ICW was calculated by subtracting the ECW from the TBW.

### Medications

Four classes of renoprotective medications were analyzed for their potential associations with the aforementioned parameters: sodium–glucose cotransporter 2 inhibitors (SGLT2is), renin–angiotensin system inhibitors (RASis), glucagon-like peptide-1 receptor agonists (GLP-1RAs), and mineralocorticoid receptor antagonists (MRAs). GLP-1RAs included both injectable and oral formulations, as well as dual glucose-dependent insulinotropic polypeptide (GIP)/GLP-1 receptor agonists. Medication intake was classified as “used” or “not used” based on medical record documentation during the observation period. Medication exposure was assessed based on prescription records. No formal assessments of medication adherence were conducted.

### Statistical analysis

All analyses were performed using SPSS version 28. For descriptive intergroup comparisons, t-test and Pearson’s correlation coefficient were employed. UACR was log-transformed prior to analysis to approximate normality. To calculate slopes, individual patient-level slopes for eGFR, log UACR, and ECW/TBW were determined via linear regression analysis (days since the first visit as the independent variable) and converted to annual changes (slope/year) by multiplying by 365. Statistical significance was defined as a two-sided *p* < 0.05. Given the exploratory nature of the study, nominal *p*-values were reported without adjustment for multiple comparisons.

To evaluate the association between ECW/TBW and eGFR slope, we constructed six prespecified multivariable linear regression models that integrated various combinations of baseline and time-averaged values for key covariates. Baseline eGFR was included in all models to account for potential confounding by kidney function, and age at baseline was used as a fixed covariate.

Renoprotective medications were not used as covariates since the primary objective was to assess the relationship between ECW/TBW and renal decline without adjusting for potential treatment effects. Linear mixed-effects models were constructed to examine the association between medication use and ECW/TBW, accounting for repeated measures within individuals.

Standardized regression coefficients (*β*) are presented for univariate linear regression analyses shown in scatter plots, whereas unstandardized coefficients (*B*) are reported for multivariable regression models.

## Results

### Baseline characteristics

The mean age of the total sample was 69.3 ± 9.7 years, and 80% of patients were male. The mean observation period was 771.4 ± 398.6 days, with an average of 8.9 outpatient visits. The mean baseline eGFR was 44.3 ± 16.6 mL/min/1.73 m^2^, and the median UACR was 1,119 mg/gCr (range 9.4–6,488.5 mg/gCr). Based on the KDIGO GFR classification, the majority of patients were categorized as G3a–G3b with severely increased albuminuria (A3). Treatment target achievement rates were modest, with 32.4% achieving blood pressure (BP) < 130/80 mmHg and 45% achieving HbA1c < 7.0%. Low-density lipoprotein cholesterol (LDL-C) targets were achieved in 67.5% of patients, whereas 37.5% had a body mass index (BMI) < 25 kg/m^2^. Regarding renoprotective medication use, 77.5% were treated with RASis, 52.5% with SGLT2is, 20% with GLP-1 RAs and MRAs were less frequently used. Heart failure was uncommon (*n* = 1), and diuretic use was rare (loop diuretic, *n* = 1; thiazide diuretic, *n*  = 1). The baseline patient characteristics, including the baseline body composition values obtained from the BIA, are summarized in Table 1.

**Table 1:** Baseline clinical characteristics of the study participants (*n* = 40)

Continuous Variables	Mean ± SD	Range
Age (years)	69.3 ± 9.7	42–88
Observation period (days)	771.4 ± 398.6	63–1,570
Number of visits	8.9 ± 3.8	3–17
eGFR (mL/min/1.73m^2^)	44.3 ± 16.6	14.2–89.1
UACR (mg/gCr)	1,119.4 ± 1,463.5	9.4–6,488.5
Log UACR (mg/gCr)	2.74 ± 0.56	0.97–3.81
Serum albumin (g/dL)	4.00 ± 0.42	2.41–4.69
HbA1c (%)	7.15 ± 0.94	5.9–10.0
Blood glucose (mg/dL)	162.2 ± 53.4	82–304
SBP (mmHg)* (n missing = 6)	138.6 ± 20.8	101–196
DBP (mmHg)* (n missing = 6)	73.6 ± 11.0	51–102
AST (IU/L)	20.5 ± 6.0	11.7–39.2
ALT (IU/L)	19.7 ± 9.6	8.3–62.9
γ-GTP (IU/L)	30.9 ± 25.2	6.8–114.5
Triglycerides (mg/dL)	192.7 ± 123.1	23.8–579.0
HDL-C (mg/dL)	59.0 ± 14.7	31.6–97.7
LDL-C (mg/dL)	98.5 ± 31.0	37.7–196.3
Serum sodium (mEq/L)	139.6 ± 2.5	132.8–146.0
BUN (mg/dL)	24.5 ± 7.5	12.0–43.4
Body composition parameters		
BMI (kg/m^2^)	25.9 ± 4.1	16.6–38.4
Skeletal muscle mass (kg)	46.2 ± 7.5	33.3–64.0
Appendicular lean mass (kg)	26.5 ± 4.8	18.3–37.0
Skeletal muscle index (kg/m^2^)	7.8 ± 1.2	5.2–11.2
Fat mass (kg)	21.6 ± 9.2	3.2–52.4
Body fat percentage (%)	28.9 ± 7.3	6.1–46.7
Total body water (L)	36.2 ± 5.9	26.0–50.3
Intracellular water (L)	21.9 ± 3.6	15.6–29.9
Extracellular water (L)	14.6 ± 2.9	10.2–25.2
ECW/TBW	0.396 ± 0.013	0.364–0.429

Categorical Variables	Category	n (%)
Sex	Male	32 (80.0%)
	Female	8 (20.0%)
ACR category	A1	1 (2.5%)
	A2	12 (30.0%)
	A3	27 (67.5%)
KDIGO-G classification	G2	6 (15.0%)
	G3a	14 (35.0%)
	G3b	13 (32.5%)
	G4	6 (15.0%)
	G5	1 (2.5%)
Control achievement, n (%)		
BP < 130/80 mmHg, n/N (%)		11/34 (32.4%)
HbA1c < 7.0%, n (%)		18 (45.0%)
LDL-C < 120 mg/dL, n (%)		27 (67.5%)
*Renoprotective medications*		
RASis use		31 (77.5%)
GLP-1 RAs use		8 (20.0%)
MRAs use		2 (5.0%)
SGLT2is use		21 (52.5%)
Diuretic Therapy		
Loop		1 (2.5%)
Thiazide		1 (2.5%)
History of Heart failure		1 (2.5%)

Descriptive statistical values for continuous variables are expressed as mean ± standard deviation (SD) and ranges, unless otherwise indicated. Those for categorical variables are expressed as numbers (percentages). Body composition parameters are also expressed as mean ± standard deviation (SD) and ranges. All parameters were obtained using an InBody multifrequency bioelectrical impedance analyzer. ACR categories and KDIGO-G classification are based on the KDIGO 2022 guidelines

*eGFR* estimated glomerular filtration rate, *UACR* urinary albumin-to-creatinine ratio, *HbA1c* glycated hemoglobin A1c, *SBP* systolic blood pressure, *DBP* diastolic blood pressure, *AST* aspartate aminotransferase, *ALT* alanine aminotransferase, *γ-GTP* gamma-glutamyl transpeptidase, *HDL-C* high-density lipoprotein cholesterol, *LDL-C* low-density lipoprotein cholesterol, BUN blood urea nitrogen, *BMI* body mass index, *TBW* total body water, *ICW* intracellular water, *ECW* extracellular water, *ECW/TBW* extracellular-to-total body water ratio, ACR albumin-to-creatinine ratio, *KDIGO* Kidney Disease: Improving Global Outcomes, *RASis* renin–angiotensin system inhibitors, *GLP-1 RAs* glucagon-like peptide-1 receptor agonists, *MRAs* mineralocorticoid receptor antagonists, *SGLT2is* sodium–glucose cotransporter 2 inhibitors

*SBP and DBP were missing in 6 patients

### Univariate analyses of the associations of ECW/TBW values and log UACR with eGFR slope

**Fig. 2:**
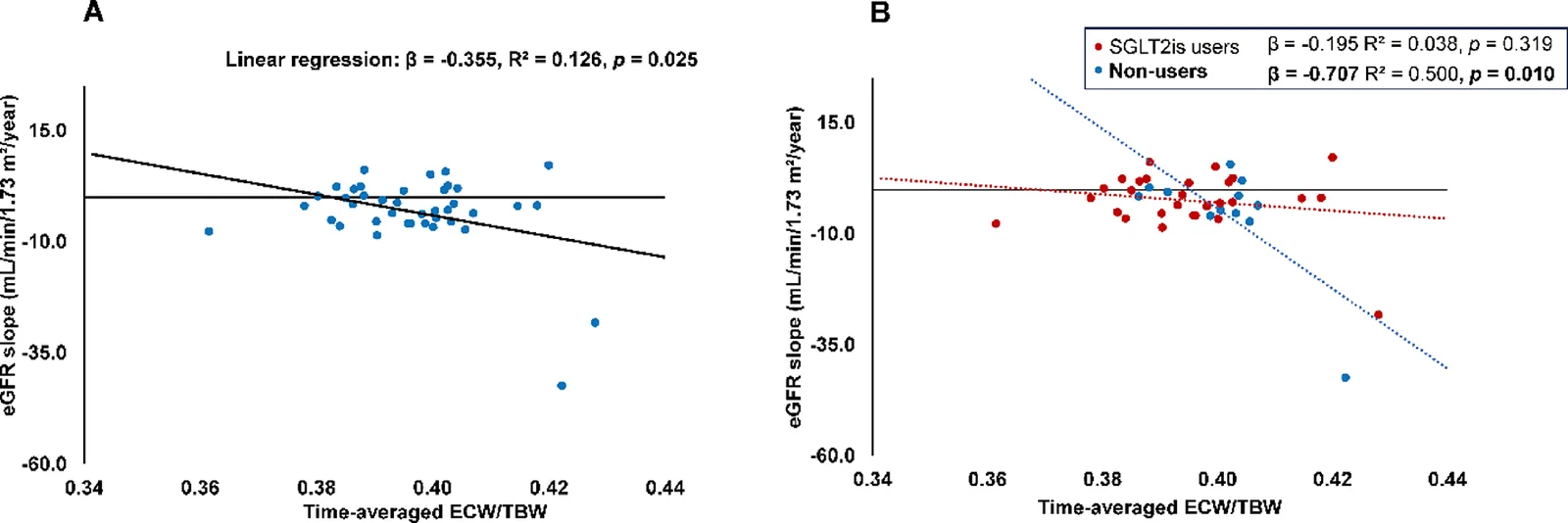
Association between time-averaged ECW/TBW and eGFR slope. **A** A significant negative correlation was observed between time-averaged ECW/TBW values and eGFR slope on univariate linear regression analysis (*β* =  − 0.355, *R*^2^ = 0.126, *p* = 0.025). **B** Distribution of SGLT2 inhibitor users and non-users within the same scatter plot. The negative association between time-averaged ECW/TBW and eGFR slope appeared markedly stronger among non-users (*β* =  − 0.707, *R*^2^ = 0.500, *p* = 0.010) than among SGLT2 inhibitor users (*β* =  − 0.195, *R*^2^ = 0.038, *p* = 0.319). *eGFR* estimated glomerular filtration rate, *ECW/TBW* extracellular water/total body water ratio, *Alt text* Scatter plot showing the relationship between time-averaged ECW/TBW and eGFR slope in patients with diabetic kidney disease

On univariate analyses, both baseline (*p* = 0.014) and time-averaged ECW/TBW values (*p* = 0.025) were significantly associated with eGFR slope (Fig. 2). Similarly, the log-transformed (*p* = 0.002) and mean (*p* = 0.004) UACR were significantly associated with eGFR slope at baseline.

To evaluate whether categorization of the eGFR slope could reveal similar associations, patients were divided into two groups (*n* = 20 each) based on the median eGFR slope (− 1.94 mL/min/1.73 m^2^/year): a higher-slope group (≥ − 1.94 mL/min/1.73 m^2^/year) and a lower-slope group (< − 1.94 mL/min/1.73 m^2^/year), each comprising 20 patients. However, no significant difference in time-averaged ECW/TBW was observed between the higher and lower eGFR slope groups (0.3988 ± 0.0143 vs. 0.3948 ± 0.0116; *p* = 0.342) (Supplementary Fig. 1).

In a sensitivity analysis excluding 7 patients with nephrotic-range proteinuria (urinary protein-to-creatinine ratio ≥ 3.5 g/gCr), the negative association between time-averaged ECW/TBW and eGFR slope was directionally maintained but no longer reached statistical significance (*β* = -0.235, *p* = 0.189), likely reflecting the reduced sample size.

In subgroup analyses stratified by SGLT2i use, the negative association between time-averaged ECW/TBW and eGFR slope appeared markedly stronger among non-users (*β* =  − 0.707, *R*^2^ = 0.500, *p* = 0.010) than among SGLT2 inhibitor users (*β* =  − 0.195, *R*^2^ = 0.038, *p* = 0.319) (Fig. 2B).

### Correlation between ECW/TBW and UACR

**Fig. 3:**
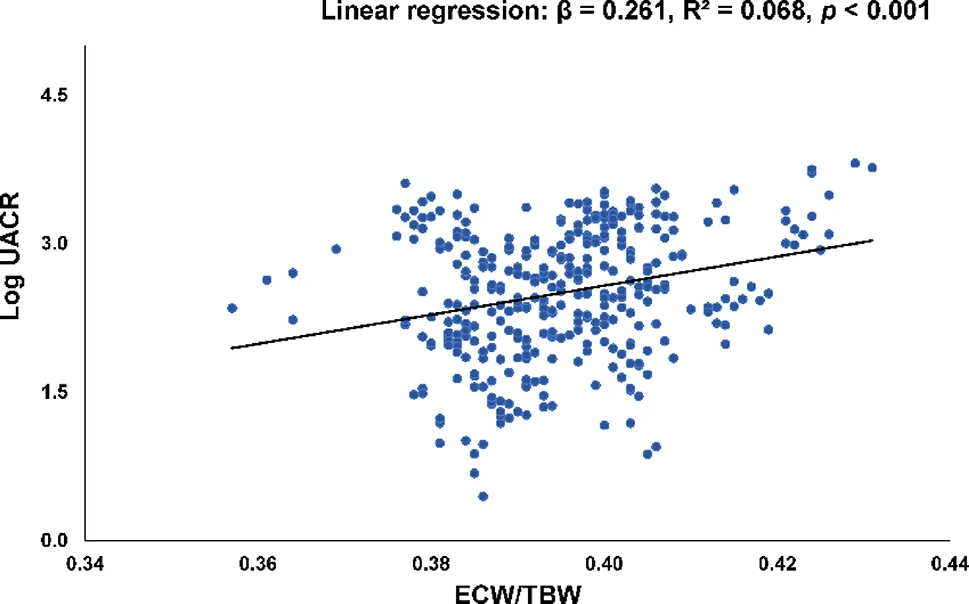
Relationship between log-transformed UACR and ECW/TBW (measurement-level). Scatter plot showing the relationship between log-transformed UACR and ECW/TBW across 355 measurement points. Linear regression exhibited a weak positive association between the two variables (*β* = 0.261, *R*^2^ = 0.068, *p* < 0.001). *UACR* urinary albumin-to-creatinine ratio,* ECW/TBW* extracellular water/total body water ratio,* Alt text* Scatter plot showing the relationship between log-transformed urinary albumin-to-creatinine ratio and ECW/TBW across multiple measurements

Univariate linear regression analysis of all measurement points (355 observations) revealed a statistically significant but weak positive correlation between ECW/TBW and log UACR (*β* = 0.261, *R*^2^ = 0.068, *p* < 0.001) (Fig. 3). A patient-level analysis with time-averaged values (*n* = 40) showed a similar but still modest trend (*β* = 0.391, *R*^2^ = 0.153, *p* = 0.013). Correlation analyses likewise indicated weak-to-modest relationships at both the measurement and patient levels (measurement-level: Pearson* r* = 0.261, Spearman  *ρ* = 0.244; patient-level: Pearson *r* = 0.391, Spearman *ρ* = 0.370).

### Multivariate models examining the correlation between ECW/TBW and eGFR slope

To examine the robustness of the correlation, six multivariate models were developed to assess different combinations of baseline and time-averaged covariates. In Model 1, baseline ECW/TBW (*B* =  − 254.1, *p* = 0.025), log UACR (*p* = 0.005), and eGFR (*p* = 0.014) values were independently associated with eGFR slope, whereas age and HbA1c were not significant predictors. In the primary model (Model 2), the time-averaged ECW/TBW was analyzed to capture chronic exposure, along with other covariates at baseline. This analysis revealed consistent results (*B* =  − 276.7, *p* = 0.019), along with significant associations for log UACR (*p* = 0.003) and eGFR (*p* = 0.016). Across Models 3–6, which incorporated various combinations of time-averaged HbA1c, log UACR, and/or SBP, the negative correlation between ECW/TBW and eGFR slope remained significant (*B* =  − 249.5 to − 275.2, *p* = 0.023–0.045) (Table 2). In the additional baseline-value model, in which systolic BP was analyzed instead of HbA1c (not shown), the association was attenuated and did not reach statistical significance (*p* = 0.299). This model included only 34 patients due to missing BP values. In an additional model based on Model 2 with further adjustment for SGLT2is use, time-averaged ECW/TBW remained independently associated with eGFR slope (*B* =  − 253.2, *β* =  − 0.383, *p* = 0.034), whereas SGLT2 inhibitor use itself was not significantly associated with eGFR slope (*β* = 0.145, *p* = 0.277). Collinearity analysis for all models revealed variance inflation factor values < 2.1, indicating that the associations were not driven by multicollinearity.

**Table 2:** Multivariate models examining the association between ECW/TBW and eGFR Slope

Model	ECW/TBW exposure	Covariates added	B (ECW/TBW)	*p* (ECW/TBW)	*p* (logUACR)	*p* (eGFR)	*p* (HbA1c)	*p* (Age)	*p* (SBP)	Adj R^2^
1	Baseline	All covariates at baseline	− 254.1	0.025	0.005	0.014	0.598	0.548	–	0.375
2 *	Time-avg	Other covariates	− 276.7	0.019	0.003	0.016	0.532	0.398	–	0.383
3	Time-avg	+ time-avg HbA1c	− 275.2	0.023	0.002	0.025	0.658	0.374	–	0.379
4	Time-avg	+ time-avg log UACR	− 259.4	0.032	0.004	0.012	0.5	0.235	–	0.368
5	Time-avg	+ time-avg HbA1c + log UACR	− 249.5	0.045	0.004	0.017	0.798	0.24	–	0.361
6	Time-avg	+ time-avg HbA1c + log UACR + SBP	− 254.3	0.041	0.002	0.017	0.447	0.197	0.24	0.368

*ECW/TBW* extracellular-to-total body water ratio, *UACR* urinary albumin-to-creatinine ratio, *HbA1c* glycated hemoglobin, *SBP* systolic blood pressure, *eGFR* estimated glomerular filtration rate, *Adj R2* Adjusted R-squared

### Association of ECW/TBW with eGFRdiff and hemoglobin levels

**Fig. 4:**
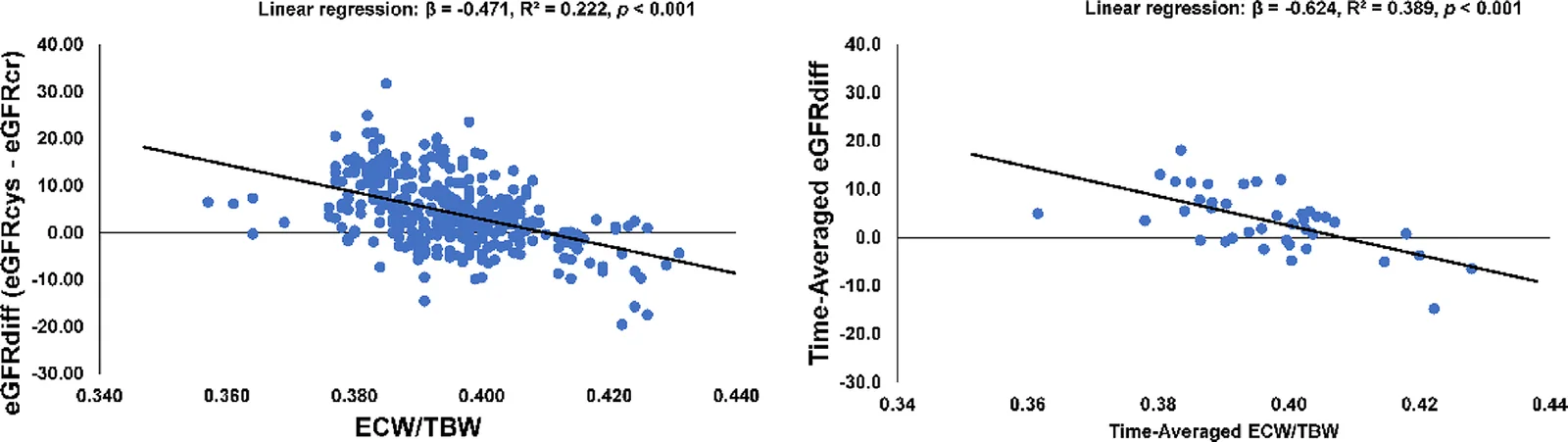
Association between ECW/TBW and eGFRdiff. Scatter plots showing the correlation between ECW/TBW and eGFRdiff (eGFRcys-eGFRcr). Linear regression analysis revealed significant associations at both the measurement level (*β* = – 0.471, *R*^2^ = 0.222, *p* < 0.001) and the patient level using time-averaged values (*β* = − 0.624, *R*^2^ = 0.389, *p* < 0.001). *ECW/TBW* extracellular water/total body water ratio

ECW/TBW was negatively associated with eGFRdiff (eGFRcys−eGFRcr). At the measurement level, linear regression demonstrated a significant association (*β* = – 0.471, *R*^2^ = 0.222, *p* < 0.001). A similar association was observed at the patient level using time-averaged values (*β* = − 0.624, *R*^2^ = 0.389, *p* < 0.001) (Fig. 4).

**Fig. 5:**
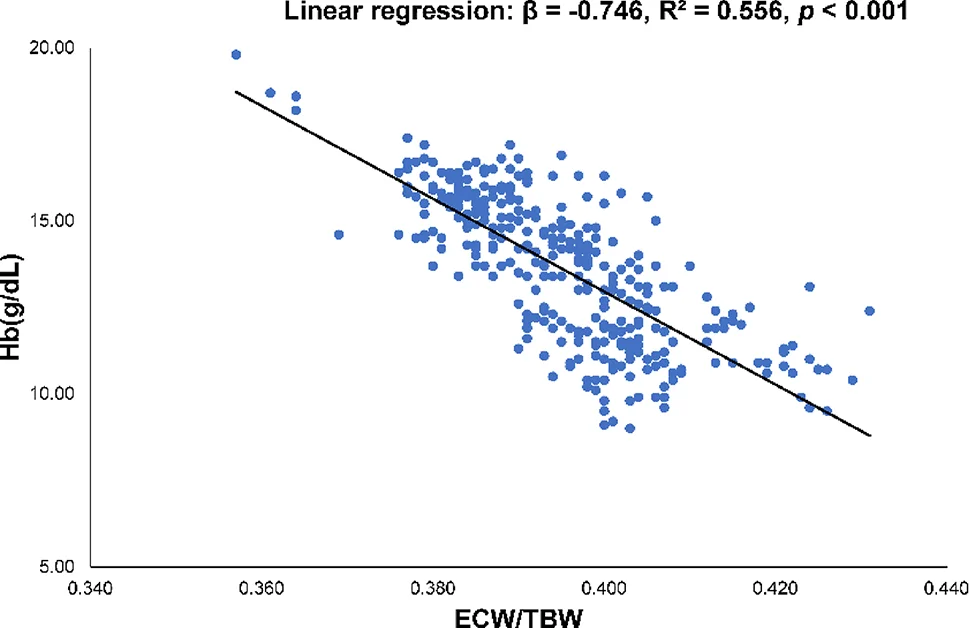
Association between ECW/TBW and hemoglobin levels. Scatter plot showing the correlation between ECW/TBW values and hemoglobin levels (g/dL) across various measurement points. Linear regression analysis revealed a significant association between the two variables (*β* =  − 0.746, *R*^2^ = 0.556, *p* < 0.001). ECW/TBW, extracellular water/total body water ratio

ECW/TBW was also strongly and inversely associated with hemoglobin levels (*β* = − 0.746, *R*^2^ = 0.556, *p* < 0.001) (Fig. 5). In an exploratory analysis, interstitial fluid volume was estimated as extracellular water (ECW) minus estimated plasma volume calculated using the Kaplan-Hakim formula, as previously described [24]. Time-averaged interstitial fluid volume was not significantly associated with eGFR slope (*r* = 0.080, *p* = 0.622).

### Relationship between the use of renoprotective medications and ECW/TBW

We examined the relationship between the use of renoprotective medications and ECW/TBW. At the measurement-point level, t-tests showed that the use of each of the four agents (SGLT2is, *p* < 0.001; RASis, *p* = 0.035; GLP-1RAs, *p* < 0.001; MRAs, *p* = 0.021) was associated with significantly lower ECW/TBW values compared with nonuse (Supplementary Fig. 2).

At the patient level, we specifically assessed whether baseline age and time-averaged ECW/TBW differed with the medication type and administration regimen. Patients were categorized as “users” if the medication was prescribed at any point during the observation period, and as “nonusers” if otherwise. Among the four medication groups, GLP-1RA users were the youngest (*p* = 0.050) and had the lowest time-averaged ECW/TBW values (*p* = 0.006). No significant differences in time-averaged ECW/TBW values were observed for the other medications (Supplementary Table 1). To account for repeated measurements, linear mixed-effects models were used to assess measurement-point-level data. In the initial model integrating only medication use, both SGLT2is (*p* < 0.001) and GLP-1RA (*p* < 0.001) were significantly associated with lower ECW/TBW values, whereas RASis and MRAs were not. Given that GLP-1RA users were younger and had lower baseline ECW/TBW values, we next evaluated whether these associations persisted after adjusting for age. When age at baseline was used as a covariate, the association between ECW/TBW and GLP-1RA use weakened and became nonsignificant. However, the association between ECW/TBW and SGLT2is was significant (*p* < 0.001). Age itself was independently associated with ECW/TBW (*p* < 0.001) (Table 3).

**Table 3:** Comparison of linear mixed-effects models with and without age adjustment

Variable	Model without age	Model with age	Interpretation
SGLT2is	*p* < 0.001	*p* < 0.001	Remained significant
GLP-1RAs	*p* < *0.001*	*p* = 0.246	Not significant in the model adjusted for age
RASis	*p* = 1.000	*p* = 0.087	Not significant in both models (trend)
MRAs	*p* = 0.812	*p* = 0.285	Not significant in both models (trend)
Age (baseline)	–	*p* < 0.001	Strong independent effect on ECW/TBW

Each model evaluated the association of ECW/TBW and the use of each medication class, with or without adjustment for baseline age

*ECW/TBW* extracellular-to-total body water ratio, *SGLT2is* sodium–glucose cotransporter 2 inhibitors, *GLP-1RAs* glucagon-like peptide-1 receptor agonists, *RASis* renin–angiotensin system inhibitor, *MRAs* mineralocorticoid receptor antagonists

When ECW/TBW values were compared across groups stratified by the number of renoprotective medications used (0–4), ECW/TBW values differed significantly among the groups (ANOVA *p* < 0.001). Post hoc analyses revealed that the overall difference observed was primarily driven by the higher ECW/TBW values in patients who received no renoprotective medications, whereas values were largely comparable among those receiving 1–4 agents (Supplementary Fig. 3).

Given the very small number of patients receiving diuretics (2 at baseline; 1 initiated during follow-up), no additional analysis was performed for diuretic therapy.

## Discussion

### Key findings

This longitudinal cohort study of patients with DKD found that persistently higher ECW/TBW values were independently associated with a rapid decline in eGFR. By leveraging serial ECW/TBW measurements and modeling both ECW/TBW and eGFR slope as continuous variables over time, our study refined and extended the current understanding of the association between ECW/TBW values and DKD progression.

### Mechanistic interpretation: volume-related stress as a complementary pathway in DKD

We hypothesized that systemic volume retention is an underrecognized pathway that contributes significantly to diabetic kidney dysfunction, potentially through congestion-related effects on renal oxygenation. In diabetes, several factors can activate fluid retention, including insulin resistance with enhanced renal sodium reabsorption [12, 25], microvascular leakage [26], and often-overlooked subclinical heart failure, such as heart failure with preserved ejection fraction or left ventricular diastolic dysfunction [13, 14]. Renal hypoxia in DKD has traditionally been attributed to microscopic mechanisms, including microangiopathy, characterized by increased tubular oxygen demand due to SGLT2 activity, and a metabolic shift toward less efficient fatty acid oxidation [27, 28]. Our findings indicate that persistent, and even subclinical, volume retention, as reflected by higher ECW/TBW values, represents an additional hemodynamic factor contributing to congestion-related hypoxia, complementing the glomerular injury pathway involved in albuminuria. Consistent with this interpretation, ECW/TBW was significantly and inversely correlated with hemoglobin levels (Fig. 5), likely reflecting hemodilution associated with extracellular volume expansion, despite the low prevalence of overt heart failure or diuretic use in our cohort.

### ECW/TBW as a distinct axis from albuminuria

The present study found that both baseline and time-averaged ECW/TBW values were associated with eGFR decline, indicating that ECW/TBW represents a persistent volume-related burden contributing to renal deterioration. Elevated baseline ECW/TBW values can identify patients at higher risk of faster renal decline. Furthermore, persistently higher values may indicate chronic exposure to subclinical volume stress. Notably, univariable analyses revealed that ECW/TBW values had only limited explanatory power for variations in log-transformed UACR at both the measurement point and patient levels. Consistent with this, correlation analyses also revealed only weak relationships at both levels of analysis. Furthermore, in multivariate models incorporating log-transformed UACR, the association between ECW/TBW values and eGFR slope remained significant, confirming that ECW/TBW contributes to renal decline independently of albuminuria. Rather than reflecting a predominantly glomerular pressure-mediated process, higher ECW/TBW values may be more characteristic of systemic or interstitial volume-related stress and may thus reflect a distinct pathway underlying deteriorating kidney function. This interpretation is consistent with prior studies showing that extracellular fluid indices can reflect physiological processes beyond simple volume loading [29].

ECW/TBW was also negatively associated with eGFRdiff (Fig. 4), a dynamic indicator previously linked to adverse outcomes in population-based cohorts [30–32], including a Japanese community-based study [33], and to risk of diabetic microvascular complications in diabetic cohorts [34, 35], possibly reflecting an underlying physiological state associated with renal vulnerability.

### Clinical implications

To analyze clinical relevance, we assessed ECW/TBW in relation to commonly used renoprotective medications. In univariate analyses, ECW/TBW values were lower among users of all four drug classes, SGLT2is, RASis, MRAs, and GLP-1RAs (Supplementary Fig. 2). However, after accounting for repeated measurements and adjusting for age in mixed-effects models, only SGLT2is use remained significantly associated with lower ECW/TBW values (Table 3). ECW/TBW values also tended to decrease with increasing number of concurrent agents (Supplementary Fig. 3); however, most pairwise groups showed no significant differences. These findings indicate a consistent association between SGLT2is therapy and lower subclinical fluid retention. Other renoprotective medications showed similar trends, although their effects appeared more modest when used individually. A previous study similarly found that patients with higher baseline ECW/TBW values experienced greater volume reduction following SGLT2i therapy [36], corroborating its potential value as a treatment-responsive marker. Collectively, these results suggest that, in addition to reducing renal oxygen demand by attenuating hyperfiltration and SGLT2 overactivity [27], SGLT2is may offer hemodynamic advantages, potentially by alleviating renal congestion and improving oxygen delivery. Interestingly, exploratory subgroup analyses suggested that the association between elevated ECW/TBW and eGFR decline was more prominent among SGLT2is non-users, whereas this relationship appeared attenuated in SGLT2is users (Fig. 2). These findings raise the possibility that SGLT2is may partially uncouple volume-related stress from renal function decline. However, this observation should be interpreted cautiously given the exploratory nature of the analysis and small subgroup size.

### Limitations

This study has several limitations. First, ECW/TBW values were measured using BIA, which is less accurate than gold-standard methods such as isotope dilution. However, standardized conditions and repeated within-subject measurements likely reduced variability. Second, the modest sample size limited statistical power and increased the risk of overfitting. Interpretation of eGFR slope in this real-world retrospective study should be approached with caution, as variability in follow-up duration and visit intervals, as well as limited measurements in some patients, may have influenced slope estimates. While these factors introduce uncertainty, they may also reflect real-world clinical settings, where rapid changes in kidney function can occur over short periods. Third, we did not directly assess renal congestion or interstitial stress, which limited our ability to confirm the contributions of the proposed mechanism. Finally, although all BIA measurements were conducted in the afternoon using the same device, ECW/TBW values may have still been affected by minor day-to-day variations.

## Conclusions

In conclusion, higher ECW/TBW values were associated with a faster decline in eGFR, independent of albuminuria, suggesting a potential contribution of volume overload to kidney function decline in DKD. Our findings also raise the possibility that SGLT2 inhibitors may attenuate this relationship. Collectively, these results highlight the potential value of ECW/TBW as a therapeutic target and monitoring marker in DKD.

## Supplementary Information

Below is the link to the electronic supplementary material.

Supplementary Figure 1. Comparison of time-averaged ECW/TBW values between patients stratified by eGFR slope. Patients were divided into two groups according to the median eGFR slope value (−1.94 mL/min/1.73 m²/year): a higher-slope group (≥ −1.94 mL/min/1.73 m²/year) and a lower-slope group (< −1.94 mL/min/1.73 m²/year). No significant difference in time-averaged ECW/TBW values was observed between the groups (p = 0.342). ECW/TBW, extracellular water/total body water ratio; eGFR, estimated glomerular filtration rate.

Supplementary Figure 2. Cross-sectional comparison of the ECW/TBW using four renoprotective medications at various measurement timepoints. Data are presented as boxplots. ECW/TBW were significantly lower in users compared to non-users for all agents (unpaired t-tests, all p < 0.05). ECW/TBW, extracellular water/total body water ratio. Alt text: Box plots comparing ECW/TBW values between users and non-users of renoprotective medications

Supplementary Figure 3. Cross-sectional comparisons of ECW/TBW by the number of renoprotective medications in use at various measurement timepoints. ECW/TBW differed significantly across all medication groups (ANOVA p < 0.01). Post hoc comparisons revealed that the between-group differences were mainly driven by higher ECW/TBW values in patients receiving no renoprotective medications, with significant differences found between agents 0 and 2 (p = 0.007), 0 and 3 (p < 0.001), and 0 and 4 (p < 0.001). The ECW/TBW values were mostly similar among patients treated with agents 1–4. ECW/TBW, extracellular water/total body water ratio; ANOVA, analysis of variance. Alt text: Box plots showing ECW/TBW values stratified by the number of renoprotective medications used

Supplementary Table 1. Differences in Baseline Age and Time-Averaged ECW/TBW Between Users and Non-Users of Renoprotective Medications. Users were defined as patients who received the medication at least once during the observation period. Abbreviations: ECW/TBW, extracellular-to-total body water ratio; SGLT2is, sodium–glucose cotransporter 2 inhibitors; GLP-1RAs, glucagon-like peptide-1 receptor agonists; RASis, renin–angiotensin system inhibitors; MRAs, mineralocorticoid receptor antagonists; Time-avg, time-averaged; n.s., nonsignificant

## Data Availability

The datasets generated and/or analyzed during the current study are available from the corresponding author upon reasonable request.

## Abbreviations

CKD: Chronic kidney disease
DKD: Diabetic kidney disease
eGFR: Glomerular filtration rate
UACR: Urinary albumin-to-creatinine ratio
ECW/TBW: Extracellular water/total body water
