# Supplementary material for: Association between extracellular-to-total body water ratio and eGFR decline in diabetic kidney disease: a longitudinal cohort study
Source: Clin Exp Nephrol. 2026 Jun 19;30(10):1457–67. doi: 10.1007/s10157-026-02905-x (PMC13582232; doi:10.1007/s10157-026-02905-x)
Supplement: Supplementary file 1 [file 10157_2026_2905_MOESM4_ESM.docx]

# **Supplementary Table 2.** Differences in Baseline Age and Time-Averaged ECW/TBW Between Users and Non-Users of Reno-Protective Medications

| Drug | Age at baseline  (Non-User vs. User) | *p*-value  (Age) | Time-avg ECW/TBW  (Non-User vs. User) | *p*-value  (ECW/TBW) | Significant Difference |
| --- | --- | --- | --- | --- | --- |
| GLP-1RAs | 72.76 ± 8.74 vs. 　66.74 ± 9.67 | 0.050 | 0.403 ± 0.012 vs. 　　　　　　　　　　　　　　0.392 ± 0.012 | 0.006 | Age: borderline  ECW/TBW: User < Non-User |
| SGLT2is | 71.00 ± 9.03 vs. 　68.57 ± 9.98 | 0.473 | 0.401 ± 0.010 vs.　　　　　　　　　　　　　　　　　　　 0.395 ± 0.014 | 0.171 | n.s. |
| RASis | 69.25 ± 8.06 vs. 　69.31 ± 9.91 | 0.991 | 0.400 ± 0.010 vs. 　　　　　　　　　　　　　　0.396 ± 0.013 | 0.657 | n.s. |
| MRAs | 70.58 ± 8.01 vs. 　66.93 ± 12.12 | 0.260 | 0.399 ± 0.013 vs.　　　　　　　　　　　　　　　 0.393 ± 0.014 | 0.136 | n.s. |
